# Review of a Study of Duloxetine for Painful Chemotherapy-Induced Peripheral Neuropathy

**Published:** 2013-09-01

**Authors:** Rita Wickham

**Affiliations:** Independent Oncology and Palliative Care Consultant

The problem of chemotherapy-induced peripheral neuropathy (CIPN) has risen to the forefront for oncology professionals, who must pay greater attention to this distressing and potentially debilitating adverse effect than they did in the past. This is because CIPN may occur and even be dose limiting after several frequently used antineoplastic agents, including platinum analogs (most notably oxaliplatin and cisplatin), taxanes (especially paclitaxel), vinca alkaloids (e.g., vincristine), bortezomib (Velcade), and thalidomide (Thalomid). Chemotherapy-induced peripheral neuropathy may also lead to numerous negative effects on activities of daily living, functioning, leisure activities, dressing, household and work activities, going barefoot or wearing shoes, and driving (Bakitas, 2007). There is little direct evidence to support the use of adjuvant analgesics (e.g., antidepressants and anticonvulsants) in the CIPN setting, although many clinicians empirically use them based on other pain management principles.

Our understanding of the possible mechanisms and progression of CIPN from different chemotherapy agents has increased, but it is far from complete. Other obstacles to managing CIPN include gaps in clinicians’ knowledge regarding recognition and grading of CIPN and the lack of universally agreed-upon, multidimensional, clinically relevant assessment tools that are also sensitive, reliable, valid, and easy to use (Farquhar-Smith, 2011). Furthermore, patients are often reluctant to report manifestations that might interfere with what they consider to be "life-saving" chemotherapy; there are few interventions to prevent, minimize, or treat CIPN.

With these quandaries in mind, it is useful to review an article by Smith and colleagues entitled "Effect of Duloxetine on Pain, Function, and Quality of Life Among Patients With Chemotherapy-Induced Painful Peripheral Neuropathy," which was recently published in the Journal of the American Medical Association (Smith et al., 2013). As the authors of this notable paper point out, this is the first publication of a large phase III study that explored a potentially effective intervention for painful CIPN caused by platinum and taxane analogs. Also of note, Dr. Smith, the first author, is an oncology nurse scientist whose clinical and research interests include CIPN, neuropathic pain, and cancer survivorship.

The authors of this article very clearly laid out in their research report the rationale for the study, the research questions, their primary and secondary endpoints, data analysis, and discussion of their findings. This approach allows the reader to "buy into" the conclusions the researchers drew from their study, which was designed to determine whether duloxetine—a selective serotonin and norepinephrine reuptake inhibitor (SNRI) antidepressant—would have usefulness in relieving neuropathic pain in adult cancer patients with confirmed CIPN. The strong study design and relatively large sample size were capable of generating trustworthy, clinically important information that supported the study hypotheses.

## Summary of the Study

A major strength of this multisite study was its design: prospective, randomized, double blind, and crossover (Smith et al., 2013). This is the type of research that can generate the highest-quality evidence upon which to base clinical decisions. The main hypothesis was that duloxetine would be superior to placebo to decrease pain after 5 weeks, in the first or the second treatment period. Secondarily, the study sought to assess the effect of duloxetine on quality of life (QOL), functioning, and adverse events.

**STUDY METHODS**

A total of 231 patients with any type and stage of cancer participated in this study. These patients had been treated at one of eight sites across the United States and were eligible to participate if they were at least 25 years old and the only chemotherapy regimen they had received included a platinum or taxane: oxaliplatin, cisplatin, paclitaxel, nanoparticle albumin-bound paclitaxel, or single-agent docetaxel (Smith et al., 2013). Eligible patients had CIPN confirmed by symptom history and symmetric stocking-glove numbness, paresthesias, or loss of deep tendon reflexes that started after receiving neurotoxic chemotherapy; at least grade 1 sensory pain (based on the National Cancer Institute [NCI] Common Terminology Criteria for Adverse Events [CTCAE] version 3.0 scale); and at least moderate (average of 4 on a scale of 0 to 10) neuropathic pain for 3 or more months after completing chemotherapy.

Patients were not eligible if they had received other neurotoxic chemotherapy agents, had received other chemotherapy before the neurotoxic agent, or were currently receiving chemotherapy. They could be taking analgesics (nonsteroidal anti-inflammatory drugs, acetaminophen, or opioids) as long as their doses were stable (no new analgesics or discontinued analgesics, with 24-hour total doses not fluctuating by > 10%) in the 2 weeks before they began the study, but they could not be taking agents known to affect serotonin levels. Patients with type 2 diabetes or peripheral vascular disease who had pain believed to be related to CIPN could participate in the study, but they were defined as "high risk." Equal numbers of high-risk patients were assigned to each treatment group to control for the potential confounding effect of comorbid illness on CIPN.

Random assignment of the 231 patients led to 115 patients in group A (duloxetine first, placebo second) and 116 patients in group B (placebo first, duloxetine second). However, 17 patients never received either treatment or did not provide data during the first treatment period, so 214 patients were evaluable. The dropout rate due to adverse effects in the initial treatment phase of the study was 11% for the duloxetine-treated patients but only 1% for the placebo-first group (* p* < .001). The actual toxicity of each patient who dropped out of the study was not provided, but Table 1 shows the grade 2/3 adverse effects experienced by at least 3% of patients in both groups.

**Table 1:**
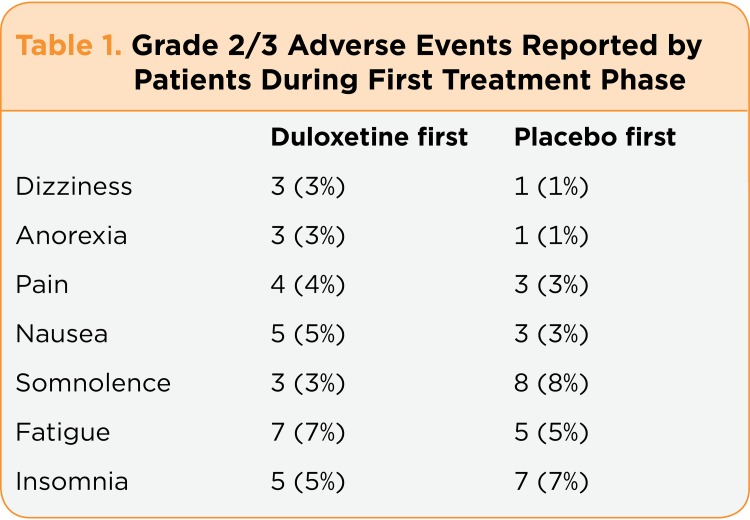
Table 1. Grade 2/3 Adverse Events Reported by Patients During First Treatment Phase

A power analysis was done, assuming that with 20% attrition, a sample size of 232 would result in 186 evaluable patients and would give the study 90% power (2-sided á of .05) to detect a 0.98-point change in average pain between the duloxetine and placebo groups. This was similar to calculations used in diabetic research on duloxetine.

Patients received identical capsules containing either duloxetine or placebo. During the first week of each treatment period, they took one capsule per day (duloxetine 30 mg or placebo); in weeks 2 through 5, they took two capsules per day (duloxetine 60 mg or placebo), which were similar doses to those given in other studies of painful peripheral neuropathy (Goldstein, Lu, Detke, Lee, & Iyengar, 2005; Sun, Zhao, Zhao, Bernauer, & Watson, 2012; Yang et al., 2012). Weeks 6 and 7 were a washout period to allow for the excretion of duloxetine, which could influence responses in the second treatment period. The study schema is shown is Table 2.

**Table 2:**
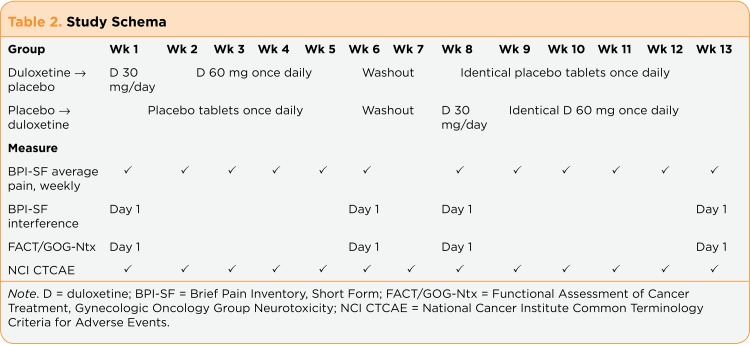
Table 2. Study Schema

**ASSESSMENT**

Measures used to assess pain, peripheral neuropathy, and QOL were the Brief Pain Inventory Short Form (BPI-SF), the NCI CTCAE, and the Functional Assessment of Cancer Treatment, Gynecologic Oncology Group Neurotoxicity (FACT/GOG-Ntx) subscale (Smith et al., 2013). The change in average pain during the initial treatment period, the main variable of interest, was measured using the BPI-SF average pain severity item on the first day of weeks 1 and 6. Average pain severity was selected based on recommendations from the Initiative on Methods, Measurement, and Pain Assessment in Clinical Trials (IMMPACT) study (Dworkin et al., 2010). The investigators also measured the proportion of patients in both groups who experienced any decrease in pain and those who had clinically significant decreases in pain severity (30% or 50%).

Pain interference with function and daily activities was measured by summing other BPI-SF items for a total interference score (Smith et al., 2013). The effect of CIPN on QOL was measured by the FACT/GOG-Ntx, which assesses numbness, tingling, and discomfort in the hands or feet; difficulty hearing; tinnitus; joint pain or muscle cramps; weakness; trouble walking or buttoning buttons; and agnosia (inability to identify small shapes placed in the hand). There were no published data defining a cut point for a clinically important change in the FACT/GOG-Ntx score, so the researchers defined a 2- to 3-point change as a clinically meaningful improvement in QOL. Patients completed the NCI CTCAE each week to assess adverse events and baseline and weekly sensory CIPN. Secondary endpoints in CIPN-related QOL were also measured by these instruments. Changes attributable to initial treatment were the differences between the week 1 and week 5 scores, and changes for crossover treatment used week 8 and week 12 scores. Analysis of covariance stratified by neurotoxic agent or risk of painful CIPN was used to test for any group effects during the initial treatment period on the primary and secondary endpoints.

**RESULTS**

The main results focused on measures at the end of the first treatment period, which are discussed (unless otherwise specified). Of greatest importance, the study found that the duloxetine-treated patients had a significantly greater decrease in average pain than the placebo-first patients (mean change scores, 1.06 vs. 0.34;* p* = .003), which was a moderately large effect size of 0.513 (Smith et al., 2013). As can be seen in Table 3, the 95% confidence intervals for duloxetine and placebo do not overlap. Changes in pain severity during the second (crossover) treatment period were also statistically significant and related to treatment (duloxetine vs. placebo; * p* < .001), but this was not an order effect *(p* =.43). That is, it was not related to whether patients received duloxetine first or second. The mean difference in the change in average pain score between the groups was 0.73 *(p* =.003), similar to differences in studies that led to US Food and Drug Administration (FDA) approval of duloxetine for painful diabetic neuropathy, fibromyalgia, and osteoarthritis (range, 0.60–0.98). Furthermore, more patients who received duloxetine (59%) had some decrease in pain than did placebo-treated patients (38%). Patients who got duloxetine were more likely to have clinically meaningful reductions in their pain (30% or 50%) than were those who got placebo (relative risk, 1.96 and 2.43, respectively). The authors concluded (based on the IMMPACT study recommendations) that 10% to 20% decreases in pain severity represent minimally clinically important findings, whereas a 30% change is a moderately important improvement and a 50% change is a substantially important improvement.

**Table 3:**
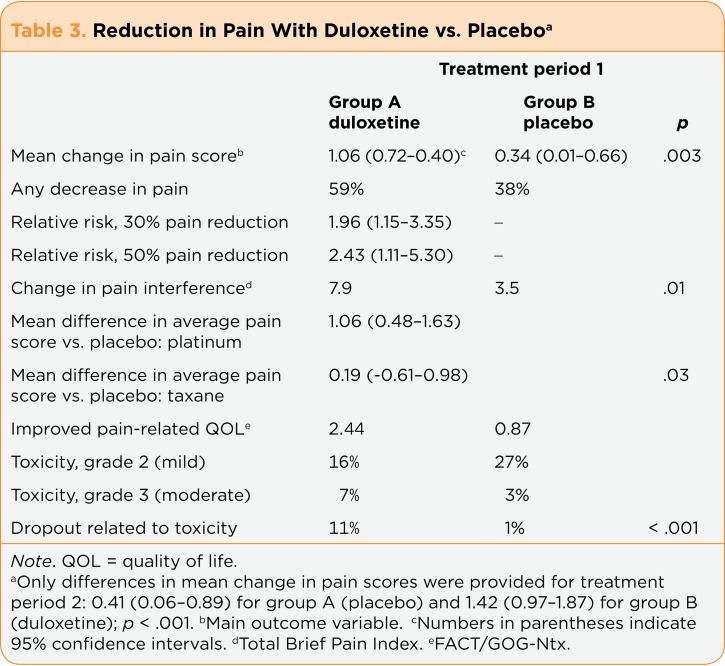
Table 3. Reduction in Pain With Duloxetine vs. Placebo

Another potentially clinically useful finding was a suggestion that duloxetine might be more beneficial for patients with CIPN secondary to platinum analogs than to taxanes (Smith et al., 2013). As compared to placebo, the relative risk of experiencing a 30% reduction in pain with duloxetine in platinum analog-treated patients was 3.05 and that for a 50% reduction was 3.78, whereas the relative risk of a 30% pain reduction was 0.97 and a 50% pain reduction was 1.22 with duloxetine among taxane-treated patients *(p* =.13). The researchers had defined the minimal clinically important difference in pain severity as a 0.98 difference in mean average pain severity between the treatment groups.

At the end of the initial treatment period, as compared to placebo, duloxetine-first patients reported a greater decrease in pain that had interfered with daily functioning *(p* =.01). The change in mean interference score for patients treated with duloxetine first was 7.9 vs. 3.5 for patients treated with placebo first. The mean difference between the two groups in mean change score was 4.40. Before starting the study, more patients who were taking other pain medications were assigned to placebo than to duloxetine: 43% vs. 31%. This difference held at the end of the first treatment period, when 36% vs. 29% were taking concomitant medications. However, 27% of patients in the duloxetine group compared with 19% of placebo-treated patients discontinued all medications by the end of the initial treatment period.

The secondary study endpoints also favored duloxetine over placebo. Pain-related QOL improved more in patients treated with duloxetine than in those given placebo. Mean changes in the FACT/GOG-Ntx total score were 2.44 for duloxetine-treated patients vs. 0.87 for placebo-treated patients *(p* =.03). The mean change score difference between the groups was 1.58 *(p* =.03). Furthermore, 41% of patients who took duloxetine and 23% of patients who received placebo reported a decrease in numbness and tingling in the feet (not significant). The trend held through the second treatment period, and 41% of patients who crossed over to duloxetine vs. 21% of patients treated with placebo had improved hand numbness and tingling.

Adverse effects did not seem to be a major problem for most patients, although they may have led patients to discontinue treatment. There were no grade 4 (moderately severe) or 5 (severe) adverse effects in either treatment group, and the frequency of grade 2 (mild) events (16% in duloxetine-treated and 27% in placebo-treated patients) and grade 3 (moderate) events (7% vs. 3%) was similar. Fatigue, insomnia, and nausea were most common in patients treated with duloxetine, whereas somnolence, insomnia, and fatigue were the most common in patients treated with placebo.

## Authors’ Conclusions

Smith and colleagues (2013) concluded that compared to placebo, 5 weeks of duloxetine was associated with a statistically and clinically significant improvement in pain and may work better for oxaliplatin-induced than taxane-induced painful CIPN. They also reminded readers that when thinking about clinical significance, they should consider treatment side effects, the rapidity of the drug effect, tolerability, and the treatment influence on other functions and QOL—all of which were positive in this study. On the other hand, the authors pointed out that the mechanisms of platinum- and taxane-induced peripheral nerve injury are different, which may explain why duloxetine seemed more efficacious for platinum-related than taxane-related neuropathic pain.

The authors recognized several limitations of their study, including the imbalance in dropout rates due to adverse effects in duloxetine- vs. placebo-treated patients (11% vs. 1%, respectively), even though the incidence of adverse effects was similar in both groups (Smith et al., 2013). They postulated that this might be partially related to the finding that more duloxetine-treated patients had grade 3 adverse events and also might have dropped out if they guessed which drug they were taking. Baseline pain determination may also have been a limitation; while oncology professionals typically use the NCI CTCAE or similar grading scales for CIPN to guide a focused history and physical examination and to grade CIPN severity, the researchers did not specifically train the examiners regarding CTCAE use because grading is "deeply embedded into oncology practice" (Smith et al., 2013, p. 1365) and the CTCAE has suboptimal interrater reliability and poor sensitivity to detect subtle changes.

## A Reviewer’s Thoughts

In reviewing research reports such as this one, a reader might think about whether it was ethical to include a placebo arm, as well as what the rationale was for comparing duloxetine to placebo at all. In addition, a reader may question whether to consider a relatively small statistically significant difference in decreased average pain scores when patients were taking duloxetine as clinically significant. And finally, should the recognized shortcomings of the CTCAE in assessing peripheral neuropathy accompanied by pain influence how we judge the study?

**INCLUSION OF PLACEBO**

There is no ethical issue in comparing duloxetine to placebo because there is no "standard" treatment—another drug or combination with confirmed efficacy—for CIPN. If there were, the research would have used this as the comparison arm. In fact, there is scant research focused on CIPN and little prior data to support specifically using duloxetine for CIPN. Two small studies were done in Asia. One, a pilot study that included only 15 cancer patients with neuropathic pain, found that 7 patients (about 47%) experienced reduced pain with duloxetine at doses of 20 to 40 mg/day (Matsuoka et al., 2012). The other study was a prospective, single-arm, open-label study that included 39 patients who experienced chronic CIPN after receiving oxaliplatin-based chemotherapy (Yang et al., 2012). Patients were scheduled to receive at least 12 weeks of duloxetine to assess its effectiveness on pain severity, neuropathic symptoms, and symptom interference with activity (measured by the CTCAE). In the first 3 weeks, 9 patients withdrew from the study because of adverse effects. Among the remaining 30 patients, 19 achieved ³ 30% pain relief. Of these, 9 had improved neuropathy.

And why implement a study with a placebo arm at all if there is any other potentially useful drug—in this case, perhaps pregabalin—that has FDA approval for painful diabetic neuropathy? Numerous (usually unpublished) studies have had "negative results," that is, a drug or compound is found to be no better than placebo, thereby preventing FDA approval of a new drug or use of an approved agent for a new purpose. Placebo arms, particularly in double-blind studies, have been important since Beecher, a renowned anesthesiologist, published his classic article proposing that placebos could improve or reverse various conditions in approximately 35% of patients (Beecher, 1955). For instance, Chvetzoff & Tannock (2003) reviewed a total of 37 randomized, placebo-controlled studies of cancer patients and found that placebo treatment was associated with moderate rates of improvement in pain, appetite, weight, and performance status as well as rare to low (2% to 7%) tumor response.

The point of the placebo arm is to separate out positive psychosocial influences (such as a supportive and trusting relationship with an advanced practitioner, nurse, or physician) and expectations on treatment outcomes (placebo effects) from actual physical effects (Watson, Power, Brown, El-Deredy, & Jones, 2012). Negative influences are called "nocebo" effects. The study of Smith and colleagues illustrates the power of the placebo effect: 38% of patients who initially got placebo reported some decrease in their pain, although the mean reduction was less than in patients who got duloxetine. Conversely, a placebo may be justified when the comparator drug has significant adverse effects (Nagasako & Kalauokalani, 2005). Patients in the Smith et al. study also experienced adverse effects, although more patients who got duloxetine than placebo dropped out of the study because of adverse effects. Similarly, Chvetzoff & Tannock (2003) found that toxicity or adverse effects occasionally led to treatment withdrawal from a placebo arm in studies of cancer patients.

**BENEFIT OF SELECTION BIAS**

Another interesting factor in the Smith et al. study is that the sample was limited to patients whose only previous chemotherapy included a platinum analog (oxaliplatin or cisplatin) or a taxane (paclitaxel, nanoparticle paclitaxel, or docetaxel) and who were not currently receiving any chemotherapy (which might be increasing CIPN), which eliminated the problem of intervening variables. This "selection bias" was fortuitous in that it illustrated the fact that different mechanisms of CIPN may influence effective treatment measures (and may limit generalization to other neurotoxic chemotherapy agents). It also highlights the idea that taxane-induced CIPN is a larger problem than some clinicians realize. Thus, in clinical practice, one might opt to try duloxetine in patients with CIPN associated with a taxane or other agent. Unanswered questions for using duloxetine or any adjunct agent include when to start, how to escalate doses (and what maximum dose to go to), what dose-limiting adverse effects are most important to assess, what constitutes a "successful" analgesic response, and how long a time-limited trial should go on before changing to a different adjunct agent.

**IS IT PRACTICE-CHANGING?**

Regarding the statistically significant differences of duloxetine over placebo, one might question whether the differences in mean pain changes are large enough to influence clinical decision-making regarding prescribing duloxetine to treat painful CIPN. The answer is yes: 59% of patients experienced some decrease in pain after 5 weeks of taking duloxetine, and more duloxetine-treated patients decreased their opioid analgesic intake. While a relatively small difference in mean pain (1.06 on a scale of 0 to 10) ratings would not seem impressive if two opioid analgesics were compared for nociceptive pain, neuropathic pain is notoriously difficult to control. Neuropathic pain may be somewhat responsive to opioid analgesics, but dose escalations to relief of pain are not usually possible without causing distressing adverse effects. Prescribers often empirically add an adjuvant analgesic (e.g., selective serotonin reuptake inhibitor [SSRI] or SNRI, anticonvulsant, or older antidepressant) to a patient’s analgesic regimen based on data in patients with other types of neuropathic pain.

Smith and colleagues’ study adds weight to selecting duloxetine for CIPN, and even relatively modest decreases in pain may allow lower opioid analgesic doses. Furthermore, their study confirmed that some patients also experienced improved QOL secondary to reductions in the dysesthesia and paresthesia patients describe in their feet and hands (stocking and glove distribution). It would be valuable to learn whether longer-term use would lead to greater relief of pain and symptoms reflecting nerve damage.

**IMPROVEMENT OF ASSESSMENT TOOLS**

Smith and colleagues (2013) recognized the need to identify more clinically effective and not overly burdensome means to assess baseline and interval peripheral nerve function and damage than CTCAE items (Table 4), which are currently the best we have in oncology. The CTCAE construction was consensus based, and there are no data to support the reliability and validity of the tool or of individual items (Trotti, Colevas, Setser, & Basch, 2007). Because the symptoms and severity of CIPN are largely subjective, recognition and grading are often difficult; comparing published studies is challenging because there is no universal standard for quantification of CIPN symptoms, and physicians tend to underreport CIPN incidence and severity (Kuroi et al., 2008). Furthermore, physicians, nurses, physician assistants, and patients are often unclear about the terms we have to describe neuropathic pain (e.g., neuropathy, paresthesia, dysesthesia).

**Table 4:**
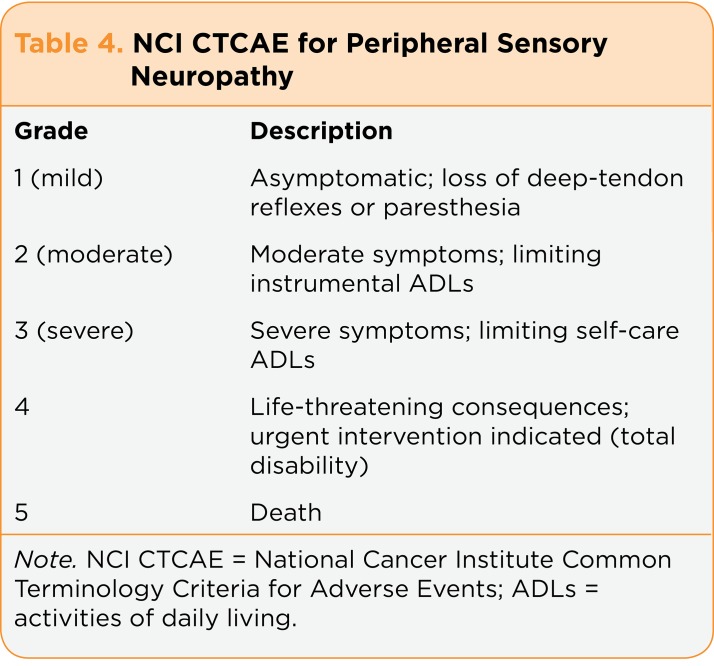
Table 4. NCI CTCAE for Peripheral Sensory Neuropathy

It is worth noting that most assessment tools have been designed to measure neuropathy from other conditions (e.g., diabetic neuropathy) and have not been validated in patients with CIPN (Stubblefield et al., 2009). One recent study confirmed that intraclass correlation coefficients of CTCAE items among triads of oncologists, oncology nurses, and patients were generally modest and might lead to questionable clinical decisions (Atkinson et al., 2012). Study participants rated 7 CTCAE items (nausea, vomiting, constipation, diarrhea, fatigue, dyspnea, and neuropathy) within 68 minutes of each other, and analysis calculated correlation coefficients ranging from 0.50 (for constipation) to 0.71 (for neuropathy). Clinically feasible and useful assessment tools are clearly needed. To this end, Smith et al. (2011) published a preliminary assessment of a treatment and referral algorithm for CIPN that advanced practitioners can consider implementing. Readers can access this article directly by scanning the barcode on page 362 or using the link provided in the reference list.

## SUMMARY 

The work of Smith et al. (2013) discussed here represents a solid study and provides us with valuable data to improve the management of our patients with CIPN. It builds upon their previous research and supports the use of duloxetine starting with a dose of 30 mg/day and escalating to 60 mg/day in 1 week. Another strength is that this study clearly identifies its limitations and suggests other important clinical questions that still need to be answered. We can look forward to further enlightening research from Smith and colleagues.
